# The effects of olive oil consumption on cognitive performance: a systematic review

**DOI:** 10.3389/fnut.2023.1218538

**Published:** 2023-10-11

**Authors:** Asra Fazlollahi, Kimia Motlagh Asghari, Cynthia Aslan, Maryam Noori, Seyed Aria Nejadghaderi, Mostafa Araj-Khodaei, Mark J. M. Sullman, Nahid Karamzad, Ali-Asghar Kolahi, Saeid Safiri

**Affiliations:** ^1^Neurosciences Research Center, Aging Research Institute, Tabriz University of Medical Sciences, Tabriz, Iran; ^2^Student Research Committee, Tabriz University of Medical Sciences, Tabriz, Iran; ^3^Physical Medicine and Rehabilitation Research Center, Aging Research Institute, Tabriz University of Medical Sciences, Tabriz, Iran; ^4^Immunology Research Center, Tabriz University of Medical Sciences, Tabriz, Iran; ^5^Student Research Committee, School of Medicine, Iran University of Medical Sciences, Tehran, Iran; ^6^Endocrinology and Metabolism Research Center, Institute of Basic and Clinical Physiology Sciences, Kerman University of Medical Sciences, Kerman, Iran; ^7^Department of Life and Health Sciences, University of Nicosia, Nicosia, Cyprus; ^8^Department of Social Sciences, University of Nicosia, Nicosia, Cyprus; ^9^Department of Persian Medicine, School of Traditional Medicine, Tabriz University of Medical Sciences, Tabriz, Iran; ^10^Nutrition Research Center, Tabriz University of Medical Sciences, Tabriz, Iran; ^11^Social Determinants of Health Research Center, Shahid Beheshti University of Medical Sciences, Tehran, Iran; ^12^Clinical Research Development Unit of Tabriz Valiasr Hospital, Tabriz University of Medical Sciences, Tabriz, Iran

**Keywords:** olive oil, Mediterranean diet, cognition, cognitive performance, geriatrics, systematic review

## Abstract

**Introduction:**

The Mediterranean diet is marked by the regular intake of olive oil, which may play a role in preventing and protecting against cognitive deterioration and dementia. The strength of these effects have been examined by several recent randomized controlled trials (RCTs), but their findings have not been consistent. In light of this inconsistency, the present study performed a systematic review to examine the relationship between the consumption of olive oil and cognition.

**Methods:**

The Web of Science, Scopus, PubMed, and Google Scholar were systematically searched up to August 11, 2023. The review included RCTs, cross-sectional studies, cohort studies and case–control studies that explored the impact of olive oil consumption on cognitive performance among those older than 55 years old. Studies were excluded if they employed a design other than those mentioned above, involved participants under 55 years old, or did not specifically examine the cognitive effects of olive oil consumption. The quality of the included studies were measured using the Cochrane risk-of-bias tool and the Newcastle Ottawa Scale checklists.

**Results:**

Eleven studies were identified, which were comprised of four cross-sectional studies, four prospective cohort studies and three RCTs. The cohort studies and RCTs consistently found that olive oil consumption had a favorable effect on cognitive performance across a number of cognitive domains over time. Similarly, all of the cross-sectional studies reported that the consumption of olive oil was positively associated with cognitive health.

**Conclusion:**

The consumption of olive oil was found to enhance cognitive functioning and to reduce cognitive decline. Further large-scale investigations are required to strengthen this conclusion.

## 1. Introduction

Human cognitive functioning rises steeply from infancy into early adulthood and then gradually declines (1). Cognitive dysfunction is commonly caused by several health problems and can result in disability and death among elderly adults (2, 3). The prevalence of cognitive impairment has increased in recent years, mostly due to increases in life expectancy and population aging (4). Moreover, the proportion of people over 60 years old is estimated to rise to almost two billion by 2050 (5). Given the limited efficacy of current medical therapies in treating age-related cognitive decline, developing effective preventative strategies is critical for reducing the burden of cognitive impairment (4).

Dietary approaches appear to be one suitable approach for slowing down age-related cognitive decline and pathological neurodegeneration (6). A number of studies have suggested that more strictly adhering to the Mediterranean diet may help lessen the likelihood of suffering from several pathological conditions, such as cardiovascular and cerebrovascular diseases, diabetes, metabolic syndrome, some types of cancers, and neurodegenerative diseases (7–9). There is also mounting evidence that shows the Mediterranean diet (MeDi) can improve cognition (10). Moreover, MeDi adherence has been associated with a reduced total mortality rate (11, 12). The MeDi dietary pattern is exemplified by a strong reliance on plant-based foods, limited intake of animal-based foods, high intake of olive oil (which is the main fat source), moderate eating of fish and the moderate consumption of wine with meals (13).

As a major component of the MeDi, the regular ingestion of extra-virgin olive oil (EVOO) is believed to play an important role in the positive effects of this dietary pattern (14). Olive oil contains natural antioxidants, such as phenolic compounds (15), which make it a food with important biological properties. The high levels of monounsaturated fatty acids (MUFAs) and polyunsaturated fatty acids (PUFAs) further contribute to its nutritional importance (16). A number of *in vitro* and *in vivo* studies suggest that frequent olive oil consumption is linked to improved cognitive functioning, indicating it may have a neuroprotective effect in preventing the development of dementia (17–19). EVOO contains secoiridoid oleuropein-aglycone, that has been found to delay cognitive decline in older individuals free from dementia (17). The phenolic compounds in EVOO protect cells from oxidative damage caused by the free radicals formed during oxygen metabolism (20). A study in mice has shown that EVOO directly improved synaptic activity, short-term plasticity, and memory, while reducing tau neuropathology (21). In addition, research findings indicate that intensive olive oil consumption is linked to a reduced risk of impaired visual memory and verbal fluency (16). Moreover, the PREDIMED-NAVARRA RCT found that individuals that had EVOO added to their diet, along with the MeDi, had better cognitive functioning than those who consumed a control diet, although there were no associations for several cognitive domains (22). In contrast, the results from a cohort study found that high olive oil intake had no protective effect against Alzheimer’s disease (AD) or memory decline. They claimed that the oleic acid, linoleic acid, and palmitic acid, which are all found in olive oil, have no role in cognitive functioning, unlike the omega-3 fatty acids docosahexaenoic acid and eicosapentaenoic acid that are found in fish oil (23, 24). Given the inconsistent findings from studies examining the impact of olive oil intake on cognitive functioning in older adults, we aimed to systematically review all clinical trials, cohort studies and cross-sectional studies to clarify this association.

## 2. Methods

The current systematic review adhered to the guidelines set forth by the Preferred Reporting Items for Systematic Reviews and Meta-Analyses (PRISMA) (25). The pre-specified protocol for the study was registered at the local university and was completed according to the proposed objectives and planned methodology.

### 2.1. Literature search

The Web of Science, Scopus, and PubMed databases were systematically searched up to August 11, 2023, without any date, language or study type restrictions. Publications reporting the impact of olive oil consumption on cognitive performance were identified using the following keywords: (“olive oil” OR “EVOO”) AND (“cognition” OR “neurocognitive disorders” OR “cognitive impairment”). In addition, in order to identify any further eligible studies, we also manually screened the first 300 hits from the Google Scholar search engine. Furthermore, we performed backward and forward citation searches of all of the studies included. A detailed breakdown of the various stages involved in the search strategy for each individual database can be found in Supplementary Table S1.

### 2.2. Study selection

The studies found both through the electronic and manual searches were all imported into EndNote 20 (Clarivate Analytics, USA). Following the removal of all duplicate records, two authors independently screened the titles and abstracts of all identified publications, in accordance with the inclusion and exclusion criteria. After excluding irrelevant papers in the initial screening, the same two authors thoroughly examined the full-texts of all retained articles, based on the predetermined inclusion and exclusion criteria. Any disagreements were resolved by consulting other authors. The PICO (population, intervention/exposure, comparison, and outcomes) framework formulated the study question as follows: (a) population: adults aged 55 years old and above; (b) intervention/exposure: olive oil consumption; (c) comparison: any exposure or intervention other than olive oil (not necessary); and (d) outcomes: cognitive performance, cognitive decline, and the development of conditions related to cognitive ability loss, such as AD.

Studies were included if they: (1) were cross-sectional studies, cohort studies, randomized controlled trials (RCTs), or case–control studies; (2) included participants aged 55 years old and above; and (3) comprehensively investigated the effects of olive oil on cognition using standardized scales to quantify cognitive performance, such as the Rey Auditory Verbal Learning Test (RAVLT) and the Alzheimer Disease Assessment Scale-Cognitive (ADAS-Cog). Studies examining different dietary patterns, including the MeDi, were only included if they separately reported the effects of olive oil consumption on cognition. We excluded studies if: (1) they were reviews, case reports, *in vitro* or animal studies; (2) they included individuals younger than 55 years old; (3) they investigated the cognitive effects of a nutrient other than olive oil; or (4) they studied the effects of olive oil on conditions other than cognitive performance.

### 2.3. Data extraction

Two reviewers carried out data extraction using predefined forms. The extracted data from each included study encompassed the following aspects: (1) the study particulars, including the first author’s name, title, country, publication year and study design; (2) basic information about the included individuals, including study population, sample size, age, sex and level of education; (3) methods of dietary and cognitive assessment; and (4) cognitive outcomes after olive oil consumption. A second author double-checked all extracted data.

### 2.4. Critical appraisal

Two reviewers independently evaluated the risk of bias and study eligibility using the Cochrane risk-of-bias tool for randomized trials – version 2 (RoB2) (26) and the Newcastle Ottawa Scale (NOS) (27) for cohort studies, cross-sectional, and case–control. In brief, the RoB2 scale categorizes each study as low, high or an unclear risk of bias (some concerns) across the following five domains: deviations from the intended interventions, randomization process, measurement of the outcome, missing outcome data, and selection of the reported results. The NOS scale appraises the quality of the studies on three areas: the comparability of the study groups; selection of the participants for each group; and the ascertainment of exposure (case–control study) or the outcome of interest (cross-sectional or a cohort study). Within the outcome, selection, and exposure categories, a study can receive a maximum of one star for each item. In contrast up to two stars can be awarded for the comparability domain.

### 2.5. Data synthesis

There was considerable heterogeneity in the population characteristics, interventions/exposures, study designs, and outcome measures across the included studies, which meant conducting a meta-analysis was not feasible. Thus, a narrative synthesis was performed in accordance with the Synthesis Without Meta-analysis (SWiM) guidelines (28). We grouped the included studies according to the study design (interventional and observational studies) and gave primacy to the interventional studies, as they have a lower risk of bias and provide more reliable evidence. Heterogeneity was not calculated.

## 3. Results

### 3.1. Study selection

**Figure 1:**
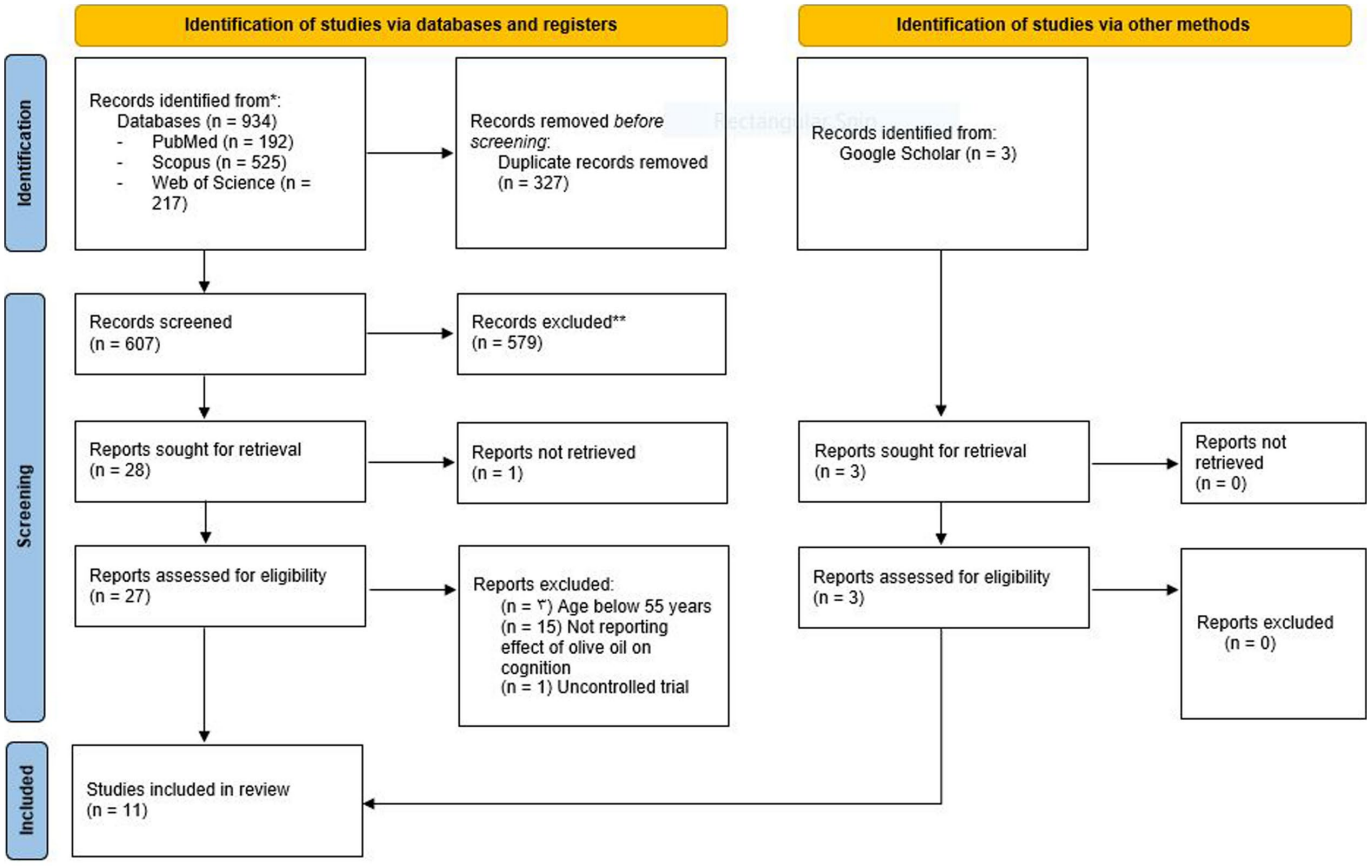
Study selection process. PRISMA 2020 flow diagram for new systematic reviews which included searches of databases, registers, and other sources.

There were 934 studies initially found in the Web of Science (*n* = 217), Scopus (*n* = 525), and PubMed (*n* = 192) searches. After removing 327 duplicate records, the title and abstract of the remaining articles were screened, which resulted in an additional 579 studies being removed. Among the remaining 28 full texts, one was inaccessible, meaning that 27 studies had their full texts assessed. Three additional articles were included following the Google Scholar search. After excluding 19 studies due to the participants being younger than 55 (*n* = 3) (29–31), not reporting the effect of olive oil on cognition (*n* = 15) (32–46), and being designed as an uncontrolled clinical trial (47), the remaining 11 studies were included in our systematic review (4, 16, 22, 23, 48–54) (Figure 1).

### 3.2. Study characteristics

The eleven included studies were comprised of four cross-sectional studies (48, 52–54), four cohort studies (4, 16, 23, 49), and three RCTs (22, 50, 51). The four cohort studies were conducted in France (16), Greece (4), Spain (49), and Germany (23), while the three RCTs were from Greece (51), Spain (22), and Italy (50). Finally, the four cross-sectional studies were conducted in Greece (48), Spain (52), Poland (53), and Morocco (54). The sample sizes ranged from 50 to 6,947 participants. In addition, the follow-up period for the RCTs and cohort studies was between one and ten years. All the cross-sectional and cohort studies used a Food Frequency Questionnaire (FFQ) for dietary assessment (Table 1). Cognitive functioning was assessed using a variety of tests that measured: attention, global cognition, processing speed, episodic memory, executive functioning, and working memory. Several tools were utilized to measure cognitive functioning, including the Alzheimer’s Disease Assessment Scale-Cognitive (ADAS-Cog) (*n* = 2), MMSE (*n* = 7), Rey Auditory Verbal Learning Test (RAVLT) (*n* = 2), and the Digit Span Memory Test (*n* = 2) (Table 2). Supplementary Table S2 presents the smoking status of the participants in the included studies, while Supplementary Table S3 provides information about their daily intake of energy and food groups. In addition, Supplementary Table S4 displays the baseline comorbidities of the participants.

**Table 1:** Baseline characteristics of the included studies.

Study ID	Study design	Country	Follow-up duration	Study Population	Sample Size	Case/Intervention, *n*	Control, *n*	Male, *n* (%)	Age, mean (SD)	Education, mean (SD), years	ApoE-ε4, *n* (%)	Diet assessment/intervention instructions	BMI, mean (SD), kg/m2	MeDi Score, mean (SD)
Anastasiou et al. 2017 (48)	Cross-sectional	Greece	No follow-up	Adults over the age of 64 years	1,803	1st quartile: 416 2nd quartile: 432 3rd quartile: 470 4th quartile: 485	No control	1st quartile: 108 (26.0) 2nd quartile: 145 (33.6) 3rd quartile: 218 (46.4) 4th quartile: 256 (52.8)	1st quartile: 73.9 (5.9) 2nd quartile: 73.3 (6.0) 3rd quartile: 72.3 (6.7) 4th quartile: 72.5 (5.8)	1st quartile: 6.7 (4.4) 2nd quartile: 7.1 (4.5) 3rd quartile: 8.1 (4.9) 4th quartile: 8.7 (4.9)	N/A	FFQ	1st quartile: 29.4 (5.0) 2nd quartile: 29.3 (5.0) 3rd quartile: 28.6 (4.4) 4th quartile: 28.7 (4.3)	N/A
Bajerska et al. 2014 (53)	Cross-sectional	Poland	No follow-up	Elderly people aged 60 years or older with high risk of metabolic syndrome, living in rural area	87	Group 1: 46 Group 2: 41	No control	Group 1: 15 (17.0) Group 2: 16 (18.0)	Group 1: 69.0 (7.0) Group 2: 72.0 (6.0)	N/A	N/A	FFQ	Group 1: 30.0 (5.5) Group 2: 29.3 (4.7)	N/A
Berr et al. 2009 (16)	Prospective cohort	France	1 to 4 years	Adults aged 65 years and over and not institutionalized	6,947	Moderate use of olive oil: 2,772 Intensive use of olive oil: 2,599	1,576	Case: moderate use: 1,099 (20.5) intensive use: 1,017 (18.9) Control: 639 (40.5)	N/A	N/A	Total: 1365 (19.7)	FFQ	N/A	N/A
Fischer et al. 2018 (23)	Prospective cohort	Germany	10 years	Urban dwelling adults (75 years or older) who sought primary care	2,622	2,622	No control	Case: 910 (34.7)	81.2 (3.4)	N/A	551 (21.0)	FFQ	25.9 (3.3)	N/A
Galbete et al. 2015 (49)	Prospective cohort	Spain	8 years	Adults over 55 years at the time of the baseline assessment	823	low MeDi score: 275 middle MeDi score: 435 high MeDi score: 113	No control	Low MeDi score: 73 Middle MeDi score: 72 High MeDi score: 74	Low MeDi score: 61.6 (6.1) Middle MeDi sore: 61.9 (5.8) High MeDi score: 62.5 (6.2)	N/A	Low MeDi score: 51 (18.6) Middle MeDi score: 89 (20.5) High MeDi score: 19 (16.8)	FFQ	1.3 (8.2)	N/A
Martínez-Lapiscina et al. 2013 (22)	RCT	Spain	6.5 years	Men (aged 55–80) and women (aged 60–80) living in the community. Free from CVD but with an elevated vascular risk	268	MeDi+EVOO: 91 MeDi+Nuts: 88	89	Intervention: MeDi+EVOO: 38 (41.8) MeDi+Nuts: 35 (39.8) Control: 47 (52.8)	Intervention: MeDi+EVOO: 67.18 (5.61) MeDi+Nuts: 67.33 (5.96) Control: 67.52 (5.67)	Intervention: MeDi+EVOO: 8.87 ± 2.08 MeDi+Nuts: 8.57 ± 2.84 Control: 8.75 (3.34)	MeDi+EVOO: 12 (13.2) MeDi+Nuts: 17 (19.3) Control: 14 (15.7)	Two interventions with MeDi (supplemented with either EVOO or a variety of nuts) contrasted against a control (low-fat diet)	Intervention: MeDi+EVOO: 28.68 (3.6) MeDi+Nuts: 28.83 (3.2) Control: 29.12 (3.5)	N/A
Mazza et al. 2018 (50)	RCT	Italy	1 year	Individuals aged 65 and older, living in the community, without debilitating disease, literate and having an MMSE score of over 20	110	55	55	N/A	Intervention: 70 (4) Control: 70 (4)	Intervention: 11 (5) Control: 11 (5)	N/A	Intervention: MeDi plus extravirgin olive oil, 20–30 g/day Control: MeDi	Intervention: 28.8 (4) Control: 28.0 (5)	Intervention: 33 (3) Control: 33 (3)
Psaltopoulou et al. 2008 (4)	Prospective cohort	Greece	6 to 13 years	Those aged 60 and above, residing in the Attica region	732	732	No Control	Case: 257 (35.1)	N/A	N/A	N/A	FFQ	N/A	Men: 5.0 (1.7) Women: 4.5 (1.6)
Tsolaki et al. 2020 (51)	RCT	Greece	12 months	Elderly patients with mild cognitive impairment aged from 60 to 80	50	Group 1 (Greek high phenolic early harvest EVOO): 18 Group 2 (Greek moderate phenolic EVOO): 16	16	Intervention: Group 1: (14.0) Group 2: 5 (10.0) Control: 3 (6.0)	Intervention: Group 1: 68.5 (6.8) Group 2: 70.8 (8.1) Control: 70.1 (6.0)	Intervention: Group 1: 11.3 (2.8) Group 2: 8.9 (3.7) Control: 10.6 (4.6)	Intervention: -Group 1: 12 (66.7) -roup 2: 12 (75) Control: 5 (31.3)	Intervention: Group 1: Greek early harvest EVOO (50 mL/day) plus MeDi guidelines Group 2: Greek EVOO (50 mL/day) plus MeDi guidelines Control: only the MeDi instructions	N/A	N/A
Valls-Pedret et al. 2012 (52)	Cross-sectional	Spain	No follow-up	Men (aged 55–80) and women (aged 60–80), living in the community, no CVD history but were diagnosed with diabetes or 3+ cardiovascular risk factors	447	447	No control	214 (47.9)	66.9	7.18	Total: 79 (17.8)	FFQ	28.5	N/A
Talhaoui et al. 2023 (54)	Cross-sectional	MOROCCO	No follow-up	Moroccan elderly subjects from 3 elderly care homes in Rabat, Kenitra, and Sidi Kacem, plus one center in Sidi kacem	151	151	No control	90 (59.6)	N/A	N/A	N/A	FFQ	N/A	Cognitively normal: 29.9 (4.4) Cognitively impaired: 28.2 (4.1)

RCT, randomized clinical trial; MeDi, Mediterranean diet; EVOO, extra virgin olive oil; CVD, cardiovascular disease; MMSE, Mini–mental state examination; FFQ, food frequency questionnaire; N/A, not available; SD, Standard deviation.

**Table 2:** Findings of the included studies.

Study ID	Olive oil consumption	Control group diet	Cognitive assessment	Outcomes	Main results
Anastasiou et al. 2017 (48)	Mean ± SD (times/day): 1.4 ± 0.5	No control	MMSE, Greek Verbal Learning Test, Verbal and Non-verbal Memory, the Boston Naming Test-short form, Medical College of Georgia (MCG) Complex Figure Test, verbal fluency, Anomalous Sentence Repetition, Benton’s Judgment of Line Orientation, Clock Drawing Test (CDT), Trail Making Test (TMT)-Part A, TMT-Part B, A gross estimate of Intellectual level and Graphical Sequence Test.	Association of olive oil intake with cognitive status (dementia, no dementia), OR (95% CI): 1.692 (0.984–2.907) Association with cognitive performance, beta±SE: −0.028 ± 0.033	Olive oil intake had no significant relationship with cognition.
Bajerska et al. 2017 (53)	Mean ± SD (serving/month) Group 1: 15.6 ± 2.74 Group 2: 20.9 ± 2.70	No control	Stroop test part A, The Spatial Span (SSP)	*Association of olive oil intake with Stroop test part A score* Regression coefficient (95% CI): −0.33 (−0.70, 0.03) beta: −0.21 *Association of olive oil intake with SSP score* Regression coefficient (95% CI): 0.06 (0.01, 0.1) beta: 0.28	The consumption of olive may provide a cognitive health benefit.
Berr et al. 2009 (16)	‘Moderate use’ (39.90%): olive oil used for cooking or dressing only, ‘Intensive use’ (37.41): olive oil used for cooking and dressing	No use of olive oil	Benton Visual Retention Test (BVRT), MMSE, Isaacs Set Test (IST)	*Adjusted longitudinal relationship between cognitive decline and olive oil consumption, OR (95% CI)* Global cognitive functioning Control: 1 Moderate use: 0.94 (0.78–1.13) Intensive use: 0.95 (0.78–1.15) Verbal fluency OR (95% CI) Control: 1 Moderate use: 0.96 (0.80–1.16) Intensive use: 0.85 (0.70–1.03) Visual memory OR (95% CI) Control: 1 Moderate use: 0.91 (0.77–1.09) Intensive use: 0.83 (0.69–0.99)	Extensive consumption of olive oil was linked to a reduced chance of cognitive deficit in visual memory and verbal fluency and visual memory. The correlation was non-significant when the MMSE was used to measure global cognitive functioning.
Fischer et al. 2018 (23)	Never: 36.3% <1 time/week: 12.2% 1 time/week: 8.2% Several times/week: 32.2% Every day: 11.1%	No control	Structured interview from the Consortium to Establish a Registry for Alzheimer’s Disease (CERAD) was used to diagnose Alzheimer’s type dementia, multi-infarct dementia and dementias of other etiology (SIDAM).	Relationship between AD incidence and olive oil consumption, HR (95% CI): 1.00 (0.93; 1.07) Relationship between memory decline and olive oil consumption, unstandardized regression coefficient (95% CI): −0.03 (−0.09; 0.04)	The investigation showed no relationship between high olive oil consumption and the risk of AD or memory decline. Unexpectedly, there was a trend for higher olive oil intake to be related to memory decline in women.
Galbete et al. 2015 (49)	Mean ± SD (g/day) Low MeDi score: 15 ± 16 Middle MeDi score: 19 ± 15 High MeDi score: 23 ± 16	No control	Telephone interview for cognitive status-modified (TICS-m)	Cognitive function changes, mean (95% CI): −0.37 (−0.68—0.06)	A higher adherence to olive oil consumption might be correlated with superior cognitive functioning.
Martínez-Lapiscina et al. 2013 (22)	The “MeDi + EVOO” group received EVOO 11/week.	Low fat diet	Rey Auditory Verbal Learning Test (RAVLT), MMSE, Clock Drawing Test (CDT), Rey-Osterrieth Complex Figure, Verbal paired associates, Trail Making Test A and B, Digit (forward and backward), Boston Naming Test, Semantic Verbal Fluency Test; Phonemic Verbal Fluency Test.	*Mean cognitive assessment scores after follow-up, by group (95% CI)* MMSE MedDiet+EVOO: 28.14 (27.72–28.56)/MedDiet+Nuts: 28.83 (27.36–28.30)/Control: 27.48 (27.07.27.90) CDT MedDiet+EVOO: 5.53 (5.19–5.86)/MedDiet+Nuts: 5.20 (4.85–5.56)/Control: 5.07 (4.72–5.42) RAVLT (immediate) MedDiet+EVOO: 32.90 (31.06–34.74)/MedDiet+Nuts: 31.28 (29.04–33.53)/Control: 30.62 (28.61–32.63) RAVLT (delay) MedDiet+EVOO: 5.81 (5.25–6.37)/MedDiet+Nuts: 5.17 (4.53–5.81)/Control: 5.21 (4.59–5.84) VPA MedDiet+EVOO: 13.43 (12.66–14.20)/MedDiet+Nuts: 12.05 (11.25–12.84)/Control: 12.81 (12.01–13.61) ROCF (immediate) MedDiet+EVOO: 13.88 (12.49–15.27)/MedDiet+Nuts: 10.94 (9.54–12.34)/Control: 11.95 (11.47–13.03)	The long-term EVOO-rich MeDi intervention led to improved cognitive functioning, compared with the control group. However, most cognitive domains were not significantly different. Those in the EVOO-rich MeDi had less MCI than those on the control group.					ROCF (delay) MedDiet+EVOO: 13.27 (11.70–14.84)/MedDiet+Nuts: 10.83 (9.44–12.22)/Control: 11.13 (9.84–12.43) Similarities MedDiet+EVOO: 10.90 (9.78–12.03)/MedDiet+Nuts: 10.64 (9.44–11.83)/Control: 10.11 (9.19–11.03) TMT-A MedDiet+EVOO: 65.50 (58.84–72.16)/MedDiet+Nuts: 71.57 (64.59–78.55)/Control: 70.79 (63.60–77.98) TMT-B MedDiet+EVOO: 199.58 (171.48–227.69)/MedDiet+Nuts: 232.41 (206.91–257.91)/Control: 220.19 (195.51–244.88) Digit (forward) MedDiet+EVOO: 7.80 (7.38–8.22)/MedDiet+Nuts: 7.40 (7.01–7.79)/Control: 6.90 (6.55–7.24) Digit (backward) MedDiet+EVOO: 4.44 (4.03–4.86)/MedDiet+Nuts: 4.51 (4.09–4.94)/Control: 4.20 (3.91–4.49) SVFT-Animals MedDiet+EVOO: 13.05 (12.20–13.91)/MedDiet+Nuts: 12.17 (11.27–13.07)/Control: 12.16 (11.32–12.99) FVFT-FAS MedDiet+EVOO: 25.66 (23.58–27.74)/MedDiet+Nuts: 23.34 (21.14–25.54)/Control: 21.29 (19.44–23.14) BNT MedDiet+EVOO: 47.27 (45.66–48.88)/MedDiet+Nuts: 45.43 (43.69–47.17)/Control: 47.19 (45.91–48.48) ROCF (copy) MedDiet+EVOO: 29.87 (28.62–31.11)/MedDiet+Nuts: 28.13 (26.63–29.62)/Control (low-fat diet): 28.13 (26.85–29.41)	
Mazza et al. 2018 (50)	The intervention group “MeDi + EVOO” received EVOO 20–30 g/day.	MeDi alone	MMSE, ADAS-cog, Verbal Fluency (VF), Beck Depression Inventory-II (BDI-II), Instrumental Activities of Daily Living (IADL) scales and Activities of Daily Living (ADL)	*Mean change within groups (before vs. after), mean ± SD* MMSE Intervention: 24.5 (1.5) vs. 25.9 (1.3)/Control: 24.6 (1.3) vs. 25.6 (1.8) ADAS-Cog Intervention: 15.3 (5.2) vs. 12.4 (4.6)/Control: 14.0 (4.5) vs. 12.5 (3.6) ADL Intervention: 6.0 (0.1) vs. 6.0 (0.1)/Control: 6.0 (0.1) vs. 6.0 (0.1) IADL Intervention: 8.0 (0.2) vs. 8.0 (0.1)/Control: 8.0 (0.1) vs. 8.0 (0.1) VF Intervention: 23 (7) vs. 25 (6)/Control: 25 (6) vs. 25 (7) BDI-III Intervention: 13 (8) vs. 12 (6)/Control: 11 (7) vs. 11 (6)	Individuals who followed the Mediterranean Diet (MeDi) along with a small dose of extra virgin olive oil (EVOO) experienced a greater short-term improvement in cognitive function scores compared to those following the MeDi alone. EVOO, being the finest quality olive oil, is believed to potentially offer a neuroprotective benefit.
Psaltopoulou et al. 2008 (4)	Mean ± SD (g/day) Men: 52.4 ± 22.5 Women: 46.3 ± 20.3	No control	MMSE	Association between one SD more of olive oil consumption and MMSE score, regression coefficient (95% CI): 0.20 (−0.11, 0.51)	Olive oil had a positive non-significant correlation with the MMSE score.
Tsolaki et al. 2020 (51)	Group 1 received 50 mL/day Greek early harvest EVOO Group 2 received 50 mL/day Greek EVOO	MeDi	Alzheimer Disease Assessment Scale-Cognitive (ADAS-Cog), Rivermead Behavioral Memory Test-Story Immediate and Delayed recall, Trail Making Test parts A & B, MMSE, Rey Osterrieth Complex Figure Test copy and delayed recall, Wechsler Memory Scales, Digit Span Forward and Backward, Letter and Category Fluency Test, Clock-drawing Test	*Mean change within groups (before vs. after), mean ± SD* MMSE Group 1: 27.9 ± 1.8 vs. 28.8 ± 1.7/Group 2: 26.6 ± 1.3 vs. 28.0 ± 1.4/Control: 28.0 ± 1.8 vs. 28.1 ± 1.8 ADAS-Cog Group 1: 12.6 ± 4.8 vs. 9.5 ± 4.4/Group 2: 15.2 ± 3.2 vs. 10.1 ± 4.2/Control: 15.3 ± 11.6 vs. 15.3 ± 11.6 Clock Drawing Group 1: 4.1 ± 0.9 vs. 4.3 ± 0.9/Group 2: 4.5 ± 0.8 vs. 4.19 ± 1.0/Control: 4.6 ± 0.6 vs. 4.5 ± 0.9 Clock Copy Group 1: 4.6 ± 0.5 vs. 4.6 ± 0.6/Group 2: 4.6 ± 0.6 vs. 4.75 ± 0.4/Control: 4.9 ± 0.3 vs. 4.9 ± 0.3 Trail Making A Group 1: 56.9 ± 18.6 vs. 65.6 ± 36.5/Group 2: 63.9 ± 30.9 vs. 64.5 ± 32.0/Control: 58.9 ± 22.1 vs. 55.4 ± 19.6 Trail Making B Group 1: 224.7 ± 112.2 vs. 234.1 ± 127.0/Group 2: 270.7 ± 146.3 vs. 238.8 ± 118.4/Control: 215.5 ± 116.4 vs. 198.9 ± 157.3	Extended treatment with high phenolic early harvest extra virgin olive oil (HP-EH-EVOO) or moderate phenolic extra virgin olive oil (MP-EVOO) resulted in significant improvements in cognitive functioning, when compared to the MeDi alone. These improvements were observed irrespective of whether the APOE 4 gene was present.					Digit Span Forward Group 1: 6.1 ± 1.2 vs. 5.2 ± 0.7/ Group 2: 5.4 ± 1.1 vs. 5.1 ± 0.7/Control: 5.4 ± 0.8 vs. 5.6 ± 0.9 Digit Span Backward Group 1: 4.1 ± 1.3 vs. 3.9 ± 0.8/Group 2: 3.5 ± 1.0 vs. 3.5 ± 0.8/Control: 4.1 ± 1.3 vs. 4.3 ± 1.1 Logical Memory I Group 1: 12.9 ± 3.0 vs. 12.4 ± 2.2/Group 2: 11.8 ± 3.5 vs. 11.5 ± 3.6/Control: 10.2 ± 4.2 vs. 10.7 ± 4.5 Logical Memory II Group 1: 12.6 ± 2.5 vs. 12.1 ± 2.2/ Group 2: 10.9 ± 4.3 vs. 11.3 ± 4.3/Control: 9.5 ± 4.2 vs. 10.2 ± 4.8 Letter Fluency Group 1: 10.9 ± 3.8 vs. 13.3 ± 4.2/ Group 2: 10.0 ± 3.3 vs. 10.3 ± 2.5/Control: 18.6 ± 10.8 vs. 21.7 ± 13.9 Category Fluency Group 1: 17.7 ± 2.9 vs. 17.9 ± 3.3/ Group 2: 15.7 ± 2.6 vs. 15.4 ± 3.7/Control: 26.9 ± 14.5 vs. 27.5 ± 17.2	
Valls-Pedret et al. 2012 (52)	Mean (range) (mL/day) Total olive oil: 38 (0–75) Virgin olive oil: 4 (0–70)	No control	RAVLT (immediate and delayed recall)	*Association between total olive oil per 10 g/d and RAVLT (immediate recall)* Regression coefficient (95% CI): 0.755 (0.151 to 1.358) Standardized regression coefficient: 0.109 *Association between overall olive oil per 10 g/d and RAVLT (delayed recall)* Regression coefficient (95% CI): 0.163 (0.010 to 0.316) Standardized regression coefficient: 0.094	Increased consumption of both overall olive oil and virgin olive oil were correlated with enhanced memory function and global cognition.
Talhaoui et al. 2023 (54)	Number of servings per week, mean (SD): Total: 4.3 (4.9) Cognitively normal: 6.1 (4.7) Cognitively impaired: 3.4 (4.9)	No control	MMSE	Association between olive oil consumption and cognitive impairment, ORa (95% CI): 0.906 (0.823–0.997)	The consumption of olive oil served as a protective factor against cognitive deterioration.

EVOO, extra virgin olive oil; MMSE, Mini–mental state examination; MeDi, Mediterranean diet; MCI, mild cognitive impairment; AD, Alzheimer’s disease; SD, standard deviation; HR, hazard ratio; OR, odds ratio; SE, standard error; CI, confidence interval.

#### 3.2.1. Randomized clinical trials

Three RCTs met the inclusion criteria, all employing a parallel design (22, 50, 51). The RCT from Greece was conducted in 2020, with a one-year follow-up and an average participant age of 69.8 years (51). These participants were allocated into three distinct groups: Group 1 received high phenolic early harvest EVOO (*n* = 18); Group 2 received moderate phenolic EVOO (*n* = 16); and Group 3, the control group, received only instructions for the MeDi (*n* = 16) (Table 1). Group 1 performed better on almost all of the cognitive measures, when compared to groups 2 and 3. In addition, Group 2 demonstrated significant improvements in the ADAS-Cog (*Z* = −3.364, *p* = 0.001) and MMSE (*Z* = 2.534, *p* = 0.011) over the measurement period, while Group 3 either exhibited worse or similar to baseline performances in most domains (Table 2).

The RCT from Spain was conducted in 2013, which featured a follow-up duration of 6.5 years and an average participant age of 74.1 when cognitive functioning was evaluated (22) (Table 1). The participants were subdivided into the following three groups: Group 1 received MeDi+EVOO (*n* = 91), Group 2 received MeDi+Nuts (*n* = 88), and Group 3 (control group) received a low-fat diet (*n* = 89). After the trial, Group 1 exhibited superior performance on all cognitive domains measured, when compared to the control group. In addition, their performance was significantly better in the fluency and memory tasks. However, those in Group 2 were not significantly different from the control group, in terms of their post-trial cognitive test scores (Table 2). At the end of the follow-up, a diagnosis of mild cognitive impairment (MCI) was confirmed in 7.8% of the participants in Group 1, 11.8% in Group 2, and 19.3% in the control group. This study demonstrated that adhering to the MeDi+EVOO helped to protect against MCI development (adjusted odds ratio: 0.341; 95% CI: 0.120–0.969; *p* = 0.044), while a non-significant effect was found for the MeDi+NUTS (adjusted odds ratio: 0.563; 95% CI: 0.222–1,427; *p* = 0.226). This study highlights the crucial role olive oil consumption has in protecting against cognitive decline.

The RCT from Italy, which was conducted in 2018, had a follow-up duration of one year and an average participant age of 70 years old (50) (Table 1). The individuals in the treatment group received MeDi+EVOO (*n* = 55), while those in the control group received the MeDi alone (*n* = 55). The MMSE and ADAS-Cog were utilized to measure any cognitive decline over the 12-month period, along with other validated psychological and functional tests, including the Verbal Fluency (VF) and Instrumental Activities of Daily Living (IADL) scales. There were substantial improvements in the ADAS-Cog scores for both groups over the study duration, as the adjusted mean changes in the scores from baseline were −3.0 ± 0.4 and −1.6 ± 0.4 for the MeDi+EVOO and MeDi groups, respectively (*p* = 0.024). While these findings indicate that regularly consuming EVOO helps maintain cognitive health, no significant differences on any other test scores were found between the treatment and control groups (Table 2).

#### 3.2.2. Cross-sectional studies

In the study from Greece (*n* = 1803), which was conducted in 2017, the participants had a mean age of 73 ± 6 years and 41% were male (48). The study aimed to investigate the association between adherence to the MeDi, including its individual components, and cognitive health. Adherence to the MeDi was evaluated using an *a priori* score (range 0–55), which was derived from a detailed semi-quantitative FFQ (Table 1). The study concluded that there was no significant relationship between olive oil consumption and cognitive status (adjusted odd ratio: 1.692; 95% CI: 0.984–2.907; *p* = 0.057), (beta ± SE: −0.028 ± 0.033; *p* = 0.397) (Table 2).

In the 2012 Spanish study (*n* = 447), participants were on average 66.9 years old, with a range of 54.7–80.2 years old (52% women) (52) (Table 1). Using the immediate and delayed recall tasks from the RAVLT, the study suggested that higher total olive oil (regression coefficient: 0.755; 95% CI: 0.151–1.358; beta: 0.109; *p* = 0.014) and virgin olive oil consumption (regression coefficient: 0.163; 95% CI: 0.010–0.316; beta: 0.094; *p* = 0.037) were associated with improved cognitive functioning (Table 2).

In the Polish study, which focused on elderly participants (*n* = 87) with an average age of 70.0 (SD ± 6.5), dietary assessment was measured using a FFQ (53). The dietary assessment was recorded as a MeDi score, which was derived from the reported consumption frequencies of the main food groups outlined in the MeDi pyramid (Table 1). The study indicated that the use of olive oil may confer cognitive health benefits, with the results from the Stroop test part A (regression coefficient: −0.33; 95% CI: −0.70 0.03; beta: −0.21; *p* = 0.05), as well as the Spatial span examination (regression coefficient: 0.06; 95% CI: 0.01 0.1; beta: 0.28; *p* = 0.05), supporting this finding (Table 2).

A study conducted in Morocco included 151 participants aged 60 and above (54) (Table 1). Cognitive functioning was assessed using the MMSE, which categorized participants as being either normal or cognitively impaired. The study utilized a validated FFQ to evaluate MeDi adherence, including the weekly consumption of its main components, such as olive oil. While the relationship between MeDi adherence and cognitive impairment was not significant, olive oil consumption emerged as the only significant protective factor against cognitive impairment (adjusted odds ratio: 0.906; 95% CI: 0.823–0.997; *p* = 0.043) (Table 2).

#### 3.2.3. Cohort studies

In a study conducted in France (*n* = 6,947), which had a follow-up period that ranged between one and four years, all participants completed a brief baseline FFQ. The participants also underwent repeated cognitive tests, including the MMSE, to evaluate verbal fluency, visual memory and global cognitive functioning (16) (Table 1). The consumption of olive oil was reported in three categories: none (22.7%), moderate (used for either dressing or cooking, 39.9%) and intensive (used for dressing and cooking, 37.4%). At baseline, those who moderately or intensively consumed olive oil had significantly lower odds of exhibiting cognitive impairment in verbal fluency and visual memory, in contrast to non-users. However, global cognitive functioning had no significant relationship. In terms of cognitive decline over the four-year follow-up, intensive olive oil use and visual memory were significantly related (adjusted adds ratio = 0.83, 95% CI: 0.69–0.99, *p* = 0.04) (Table 2).

A cohort study in Greece included 732 participants over 60 years old who had previously taken part in the European Prospective Investigation into Cancer and Nutrition cohort study (4) (Table 1). MeDi adherence was examined using a FFQ that included around 150 commonly consumed foods and beverages. Higher daily olive oil intake was reported among men compared to women (52.4 g vs. 46.3 g). Cognitive functioning was evaluated 6 to 13 years later using the MMSE score, which revealed a non-significant association between olive oil intake (beta coefficient = 0.20, 95% CI: −0.11 to 0.51, *p* = 0.204) and cognitive functioning (Table 2).

In a prospective cohort study involving 823 Spanish adults with an average age of 62 ± 6 SD, a 136-item validated FFQ was utilized to measure MeDi adherence at baseline, and those taking part in the study were categorized into low, moderate, and high consumers, based upon their MeDi score (49) (Table 1). Cognitive functioning was assessed twice at follow-up, with mean follow-up times of six and eight years, using the Telephone Interview of Cognitive Status-modified (TICS-m). Participants with low or moderate olive oil consumption demonstrated a larger cognitive decline over the measurement period than those with higher consumption (adjusted difference: −0.37; 95% CI: −0.68 to 0.06; *p* = 0.020) (Table 2).

In the study from Germany, participants with an average age of 81.2 ± 3.4 SD were regularly followed up over a 10-year time period (23). In the course of the follow-up, 418 incident cases of AD were observed in the study population (*n* = 2,622). However, the study did not find a significant relationship between higher olive oil intake and a lower risk of AD (hazard ratio:1.00;95% CI: 0.93–1.07; *p* = 0.969) or memory decline (beta: −0.03; 95% CI: −0.09 to 0.04; *p* = 0.388) (Table 2).

### 3.3. Quality assessment

In relation to the cohort studies, the risk of bias scores ranged from 6 to 8 and had an average of 6.25. Although none of the cohort studies achieved a perfect score (9/9), one study received a 6 (49), one received a 7 (16), and two studies achieved an overall score of 8 (4, 23). Ascertainment of exposure was the lowest scoring criterion (0/4, 0.0%) (Supplementary Table S5).

Among the four cross-sectional studies, the average was 7.5. Two cross-sectional studies received an overall score of 7 (53, 54) and the other two received scores of 8 (48, 52). The lowest scores were for sample size justification and the comparability between the respondents and non-respondents (both 0/4, 0.0%) (Supplementary Table S6).

Two of the three RCTs were categorized as having a low risk (22, 51), while the last had a high risk of bias (50). The selection of the reported results and the measurement of the outcome were both low risk in all three RCTs (Supplementary Table S7).

## 4. Discussion

The objective of the current systematic review was to investigate whether olive oil consumption can help protect against cognitive decline among the elderly. The review analyzed four cross-sectional studies, four cohort studies, and three RCTs. Despite some heterogeneity in the findings, the results of the 11 studies were reasonably consistent. The findings from the RCTs indicated that the consumption of olive oil could increase cognitive performance in almost all cognitive domains measured. For example, Tsolaki et al. found that the intake of Greek high phenolic early harvest EVOO and moderate phenolic EVOO, along with MeDi adherence, were correlated with superior cognitive performance. Particularly noteworthy improvements, in comparison to the control group, were found in global cognition, letter fluency, and MCI stability after one year (51). Similarly, Martínez-Lapiscina, et al. found a negative relationship between cognitive impairment and EVOO consumption. They found that an EVOO-rich MeDi was linked with superior cognition, particularly in the fluency and visual and verbal memory domains. Furthermore, following a nutritional intervention period of 6.5 years the incidence of MCI within this group was also lower than that found in the control group (22). Moreover, Elisa Mazza et al. found a short-term improvement in cognitive functioning scores for those on the MeDi plus a low dose of EVOO, when compared to those on the MeDi alone (50).

There was substantial heterogeneity in the main findings of the included prospective cohort studies. For instance, Berr et al. found that moderate and intensive olive oil intake were linked to reduced odds of having cognitive deficits in visual memory and verbal fluency. In addition, over the 4-year follow-up period those in the intensive olive oil consumption group had a significantly lower decline in visual memory, but not in verbal fluency (16). Similarly, Galbete et al. demonstrated the neuroprotective effects of olive oil intake, as well as adherence to the MeDi, on cognitive decline (49). In contrast, Psaltopoulou found that adherence to the MeDi and the consumption of olive oil had a non-significant association with cognitive functioning (4). They suggested that their unexpected findings, which are in contrast to previous results, could be caused by their relatively small sample size (*n* = 732). Furthermore, Fischer et al. did not find any relationship between high olive oil consumption and the incidence of memory decline or AD. They even suggested that increased olive oil intake may be associated with a more pronounced decline in memory among women (23). In line with the findings from the cohort studies, there was a high degree of heterogeneity in the cross-sectional studies. For example, Anastasiou et al. reported that olive oil consumption and cognition were not significantly associated (48). Nevertheless, the three remaining cohort studies all reported olive oil intake to be beneficial for cognitive health (52–54).

Many researchers have examined the effects that the MeDi and its major components, such as olive oil, have in the inhibition of neurodegenerative diseases. Olive oil is rich in a variety of phenolic compounds, including oleuropein-aglycon, which is considered to have a neuroprotective effect against cognitive decline (19, 50, 55–57). Furthermore, the positive impact of olive oil intake on neurological disorders have been comprehensively studied, in terms of the different cellular pathways and mechanisms (17). One of these is via a rise in the antioxidant content of low-density lipoproteins, coupled with its nutrigenomic effects (58).

Among olive oils, EVOO is the highest quality variant, and is made through cold mechanical extraction from olives without the use of solvents or other refining methods, which results in the preservation of the most potent antioxidants and anti-inflammatory components (17). The extraction or refining of olive oil produces an inferior quality oil. Refined olive oil, in comparison to EVOO, offers less protection against oxidative lipid damage, free radical formation, and inflammatory responses. Many studies have also suggested that EVOO consumption has no negative consequences, like apoptosis or neurodegeneration (50, 59–61). The protective effects of EVOO are thought to be more prominent in the first decades of life, highlighting the importance of initiating EVOO consumption before puberty and maintaining it throughout the individual’s life (62, 63).

While no previous systematic review has studied how cognition is affected by olive oil consumption, several published articles have systematically examined the association between MeDi adherence and cognitive health. In a systematic review and meta-analysis, Singh et al. suggested that higher MeDi adherence was linked to a lower chance of developing MCI and AD. However, they only included five studies in their quantitative analysis (64). Furthermore, in another systematic review by Petersson et al. they aimed to provide an updated understanding of the effect of the MeDi on cognitive functioning, cognitive impairment, AD, and all other types of dementia. Their review included 32 studies, which consisted of five RCTs and 27 observational studies. The majority of the studies found that MeDi adherence was linked to improvements in cognitive functioning, decreased cognitive impairment, and lowered susceptibility to dementia or AD. However, three of the studies included in the review reported no relationship between the MeDi and AD, three studies reported no correlation between the MeDi and cognitive impairment, and five studies found no relationship between the MeDi and cognitive functioning. However, there was substantial heterogeneity in these studies, in terms of the methods used to evaluate cognitive functioning, the measurement of the MeDI, as well as the study quality. The authors concluded that MeDi adherence was correlated with superior cognitive performance (65). In another systematic review, they found an approximately linear relationship between the MeDi score and the likelihood of developing a cognitive disorder. They concluded that higher MeDi scores were negatively correlated with the development of mental and cognitive disorders. However, their analysis of the dose–response found there was a non-significant linear relationship between the incidence risk of cognitive degeneration and the MeDi score (66). In addition, García-Casares and colleagues showed that stricter MeDi adherence was correlated with a significantly reduced risk of MCI and AD. In their systematic review and meta-analysis, they firstly performed a qualitative analysis and comprehensive update of the studies in this field, from a clinical view point. In the second step, they carried out a quantitative analysis to examine the impact of the MeDi on the risk of developing MCI or AD (67). Their meta-analysis of RCTs revealed that the MeDi enhanced global cognition, working memory, and delayed recall, but not verbal fluency, episodic memory, paired associates, immediate recall, processing speed, or attention. However, the strongest support was for the finding that the MeDi had a positive effect on the global cognition of older adults (68).

The present study is the most up-to-date systematic review to examine the impact of olive oil consumption on cognitive health among the elderly. Nevertheless, it’s important to acknowledge certain limitations in our study. Firstly, due to the considerable heterogeneity in study designs and reported outcomes among the included studies, we were unable to conduct a meta-analysis or sub-group analyses. Secondly, although we thoroughly searched the online databases and grey literature, there is a possibility that we missed some eligible studies, thus necessitating a cautious interpretation of our findings. Thirdly, some of the studies we included did not report all of the necessary information about their participants, especially regarding the daily intake of the different food groups. Fourthly, it should be noted that the scales utilized to measure the cognitive status of participants were not identical, which made comparing the findings challenging. Another potential limitation of our study concerns the validity and reliability of the FFQs used to assess olive oil intake in the included cross-sectional and cohort studies, since the FFQs were self-reported. In particular, elderly individuals with cognitive impairments might be less able to report their precise food intake, which could cause bias. Moreover, the included studies reported the amount of olive oil consumption in different ways and using different units, which made comparisons difficult. In addition, the study protocol was not registered in any international registries, such as the International prospective register of systematic reviews (PROSPERO), may raise the potential for bias and unintended duplication. Finally, the limited number of RCTs and the considerable risk of bias in half of them is another limitations of our study. Therefore, additional RCTs are needed in order to conclusively demonstrate the casual relationship between olive oil intake and reduced cognitive impairment.

## 5. Conclusion

This research found a positive correlation between the intake of olive oil and improved cognitive functioning among elderly adults. Furthermore, regular olive oil intake has the potential to lower the risk of cognitive decline over time. Thus, the regular consumption of olive oil is a highly recommended means to improve cognitive functioning and to prevent or delay the occurrence of cognitive disorders. Nevertheless, to gain a precise understanding of how olive oil impacts various facets of cognition, it is imperative to conduct additional observational studies, interventions, and meta-analyses.

## Data availability statement

The original contributions presented in the study are included in the article/Supplementary material, further inquiries can be directed to the corresponding authors.

## Author contributions

MA-K, A-AK, and SS conceptualized the topic. CA searched the databases. AF and KMA performed screening, full-text review, and quality assessment. AF and CA performed data extraction. AF, KMA, MN, and CA prepared the first draft of the manuscript. AF, SAN, MA-K, MJMS, NK, A-AK, and SS critically revised and edited the manuscript. SS and A-AK supervised this project. All authors reviewed and approved the final version of the manuscript.

## Abbreviations

AD: Alzheimer’s disease
ADAS-Cog: Alzheimer Disease Assessment Scale-Cognitive
CDR: Clinical dementia rating
CI: Confidence interval
EVOO: Extra-virgin olive oil
FFQ: Food frequency questionnaire
MCI: Mild cognitive impairment
MeDi: Mediterranean diet
MMSE: Mini–mental state examination
MUFA: Monounsaturated fatty acid
NOS: Newcastle Ottawa Scale
OR: Odds ratio
PRISMA: Preferred Reporting Items for Systematic Reviews and Meta-Analyses
RCT: Randomized controlled trial
RoB2: Risk of bias 2
TICS-m: Telephone Interview of Cognitive Status-modified
