# Supplementary material for: The effects of olive oil consumption on cognitive performance: a systematic review
Source: Front Nutr. 2023 Oct 11;10:1218538. doi: 10.3389/fnut.2023.1218538 (PMC10598670; doi:10.3389/fnut.2023.1218538)
Supplement: Supplementary file 1 [file Data_Sheet_1.docx]

**The effects of olive oil consumption on cognitive performance: A systematic review**

**Table S1.** Search strategy used with the four databases.

| **Database**  **(search date)** | **Step** | **Search strategy** | **Number of results** |
| --- | --- | --- | --- |
| PubMed  (8.11.2023) | #1 | "olive oil"[MeSH Terms] OR "olive oil"[Other Term] OR "olive oil"[Title/Abstract] OR "Extra virgin olive oil"[Title/Abstract] OR "Extra virgin olive oil"[Other Term] OR "EVOO"[Other Term] OR "EVOO"[Title/Abstract] | 13,629 |
|  | #2 | "cognition"[MeSH Terms] OR "cognition"[Other Term] OR "cognition"[Title/Abstract] OR "cognition disorders"[Title/Abstract] OR "cognition disorders"[MeSH Terms] OR "cognition disorders"[Other Term] OR "neurocognitive disorders"[Other Term] OR "neurocognitive disorders"[MeSH Terms] OR "neurocognitive disorders"[Title/Abstract] OR "Alzheimer disease"[Title/Abstract] OR "Alzheimer disease"[MeSH Terms] OR "Alzheimer disease"[Other Term] OR "Huntington disease"[Other Term] OR "Huntington disease"[MeSH Terms] OR "Huntington disease"[Title/Abstract] OR "cognitive dysfunction"[Title/Abstract] OR "cognitive dysfunction"[MeSH Terms] OR "cognitive dysfunction"[Other Term] OR "dementia"[Other Term] OR "dementia"[MeSH Terms] OR "dementia"[Title/Abstract] OR "cognitive decline"[Title/Abstract] OR "cognitive decline"[Other Term] OR "cognitive impairment"[Other Term] OR "cognitive impairment"[Title/Abstract] | 617,571 |
|  | #3 | #1 AND #2 | 192 |
| Scopus  (8.11.2023) | #1 | TITLE-ABS-KEY ( "olive oil" OR "Extra virgin olive oil" OR “evoo” ) | 31,417 |
|  | #2 | TITLE-ABS-KEY ( “cognition” OR "cognition disorders" OR "neurocognitive disorders" OR "Alzheimer disease" OR "Huntington disease" OR "cognitive dysfunction" OR dementia OR "cognitive decline" OR "cognitive impairment" ) | 882,662 |
|  | #3 | #1 AND #2 | 525 |
| Web of Science  (8.11.2023) | #1 | TS=("olive oil" OR "Extra virgin olive oil" OR “EVOO”) | 28,377 |
|  | #2 | TS=(“cognition” OR "cognition disorders" OR "neurocognitive disorders" OR "Alzheimer disease" OR "Huntington disease" OR "cognitive dysfunction" OR “dementia” OR "cognitive decline") | 456,381 |
|  | #3 | #1 AND #2 | 217 |
| Google Scholar  (8.11.2023) | #1 | ("olive oil" OR "Extra virgin olive oil" OR “EVOO”) AND (“cognition” OR "cognition disorders" OR "neurocognitive disorders" OR "cognitive dysfunction" OR dementia OR "cognitive decline" OR "cognitive impairment") | 18,500 |

**Table S2.** Smoking status of participants in the included studies, n (%).

| **Study ID** | **Never** | **Former** | **Current** |
| --- | --- | --- | --- |
| Anastasiou et al. 2017 (1) | N/A | N/A | N/A |
| Bajerska et al. 2017 (2) | N/A | N/A | Group 1: 5 (10.9)  Group 2: 6 (14.6) |
| Berr et al. 2009 (3) | Total: 4230 (60.9) | Total: 2339 (33.7) | Total: 378 (5.4) |
| Fischer et al. 2018 (4) | Total: 1307 (49.8) | Total: 1125 (42.9) | Total: 190 (7.3) |
| Galbete et al. 2015 (5) | N/A | Low MeDi score: 136 (49.5)  Middle MeDi score: 231 (53.1)  High MeDi score: 58 (57.3) | Low MeDi score: 41 (14.9)  Middle MeDi score: 55 (12.6)  High MeDi score: 14 (12.4) |
| Martínez-Lapiscina et al. 2013 (6) | N/A | MeDi+EVOO: 16 (17.6)  MeDi+Nuts: 18 (20.5)  Control: 23 (25.8) | MeDi+EVOO: 13 (14.3)  MeDi+Nuts: 9 (10.2)  Control: 20 (22.5) |
| Mazza et al. 2018 (7) | N/A | N/A | Intervention: 20 (36)  Control: 29 (52) |
| Psaltopoulou et al. 2008 (8) | Men: 78 (30.4)  Women: 352 (74.1) | Men: 179 (69.6)  Women: 123 (25.9) | |
| Tsolaki et al. 2020 (9) | N/A | N/A | N/A |
| Valls-Pedret et al. 2012 (10) | N/A | N/A | Total: 72 (16.1) |
| Talhaoui et al. 2023 (11) | N/A | N/A | N/A |

Abbreviations: MeDi: Mediterranean diet; EVOO: extra virgin olive oil; N/A: not available.

**Table S3.** Daily intake of energy and food groups of participants of the included studies, mean (SD).

| **Study ID** | Anastasiou et al. 2017 (1) | Bajerska et al. 2017 (2) | Berr et al. 2009 (3) | Fischer et al. 2018 (4) | Galbete et al. 2015 (5) | Martínez-Lapiscina et al. 2013 (6) | Mazza et al. 2018 (7) | Psaltopoulou et al. 2008 (8) | Tsolaki et al. 2020 (9) | Valls-Pedret et al. 2012 (10) | Talhaoui et al. 2023 (11) |
| --- | --- | --- | --- | --- | --- | --- | --- | --- | --- | --- | --- |
| **Energy Intake, kcal/d** | 1st quartile: 1708 (501)  2nd quartile: 1869 (526)  3rd quartile: 1932 (519)  4th quartile: 2022 (507) | N/A | N/A | N/A | low MeDi score: 2094 (668)  middle MeDi score: 2300 (652)  high MeDi score: 2405 (553) | MeDi+EVOO: 2334 (531)  MeDi+Nuts: 2246 (446)  Control: 2202 (485) | Intervention: 1814 (376)  Control: 1880 (452) | Men: 2141 (607)  Women: 1796 (527) | N/A | Total: 2362 | N/A |
| **Fruits, g/d** | N/A | N/A | N/A | N/A | low MeDi score: 283 (287)  middle MeDi score: 456 (284)  high MeDi score: 590 (285) | N/A | Intervention: 345 (206)  Control: 333 (162) | N/A | N/A | Total: 470 | N/A |
| **Vegetables, g/d** | N/A | N/A | N/A | N/A | low MeDi score: 413 (378)  middle MeDi score: 602 (375)  high MeDi score: 712 (376) | N/A | Intervention: 290 (136)  Control: 250 (133) | N/A | N/A | Total: 406 | N/A |
| **Fish & Seafood, g/d** | N/A | N/A | N/A | N/A | low MeDi score: 96 (71)  middle MeDi score: 122 (71)  high MeDi score: 161 (71) | N/A | Intervention: 73 (64)  Control: 61 (45) | Men: 26.2 (16.3)  Women: 23.9 (14.9) | N/A | Total: 114 | N/A |
| **Meat and meat products, g/d** | N/A | N/A | N/A | N/A | low MeDi score: 174 (61)  middle MeDi score: 154 (66)  high MeDi score: 109 (66) | N/A | Intervention: 75 (48)  Control: 79 (44) | N/A | N/A | Total: 89 | N/A |
| **Alcohol, g/d** | N/A | N/A | N/A | N/A | Low MeDi score: 9 (13)  Middle MeDi score: 10 (13)  High MeDi score: 10 (13) | MeDi+EVOO: 16 (24)  MeDi+Nuts: 11 (15)  Control: 14 (19) | Intervention: 5 (8)  Control: 9 (12) | N/A | N/A | Total: 4 | N/A |
| **Dairy, g/d** | N/A | N/A | N/A | N/A | low MeDi score: 227 (169)  middle MeDi score: 133 (168)  high MeDi score: 52 (169) | N/A | Milk  -Intervention: 122 (108)  -Control: 139 (112)  Cheese  -Intervention: 66 (50)  -Control: 48 (39) | N/A | N/A | Total: 359 | N/A |
| **Cereals, g/d** | N/A | N/A | N/A | N/A | low MeDi score: 87 (73)  middle MeDi score: 108 (73)  high MeDi score: 118 (73) | N/A | Intervention: 200 (82)  Control: 205 (88) | N/A | N/A | Total: 252 | N/A |
| **Legumes, g/d** | N/A | N/A | N/A | N/A | low MeDi score: 20 (20)  middle MeDi score: 25 (20)  high MeDi score: 29 (17) | N/A | Intervention: 25 (31)  Control: 15 (13) | N/A | N/A | Total: 19 | N/A |

Abbreviations: MeDi: Mediterranean diet; EVOO: extra virgin olive oil; N/A: not availabl

**Table S4.** Baseline comorbidities of participants in the included studies, n (%).

| **Study ID** | Anastasiou et al. 2017 (1) | Bajerska et al. 2017 (2) | Berr et al. 2009 (3) | Fischer et al. 2018 (4) | Galbete et al. 2015 (5) | Martínez-Lapiscina et al. 2013 (6) | Mazza et al. 2018 (7) | Psaltopoulou et al. 2008 (8) | Tsolaki et al. 2020 (9) | Valls-Pedret et al. 2012 (10) | Talhaoui et al. 2023 (11) |
| --- | --- | --- | --- | --- | --- | --- | --- | --- | --- | --- | --- |
| **Hypertension** | N/A | N/A | Total: 3903 (56.1) | N/A | N/A | MeDi+EVOO: 68 (74.7)  MeDi+Nuts: 70 (79.5)  Control: 71 (79.8) | Intervention: 28 (51)  Control: 29 (52) | Men: 180 (70.0)  Women: 283 (59.6) | N/A | Total: 336 (75.2) | N/A |
| **Diabetes** | N/A | N/A | Total: 645 (9.2) | N/A | N/A | MeDi+EVOO: 29 (31.9)  MeDi+Nuts: 30 (34.1)  Control: 29 (32.6) | Intervention: 23 (41)  Control: 30 (54) | Men: 36 (14.0)  Women: 65 (16.7) | N/A | Total: 250 (55.9) | N/A |
| **Hypercholesterolemia** | N/A | N/A | Total: 5153 (74.1) | Total: 1408 (53.7) | N/A | N/A | N/A | N/A | N/A | N/A | N/A |
| **Mild cognitive impairment** | N/A | Group 1: 20 (43.4)  Group 2: 4 (9.7) | N/A | Total: 436 (16.6) | N/A | MeDi+EVOO: 7 (7.8)  MeDi+Nuts: 10 (11.8)  Control: 17 (19.3) | N/A | N/A | Total: 50 (100) | N/A | N/A |
| **Dementia** | 1st quartile: 133 (32)  2nd quartile: 151 (35)  3rd quartile: 94 (20)  4th quartile: 68 (14) | N/A | N/A | N/A | N/A | MeDi+EVOO: 1 (1.1)  MeDi+Nuts: 3 (3.4)  Control: 1 (1.1) | N/A | N/A | N/A | N/A | N/A |
| **Hyperlipidemia** | N/A | N/A | N/A | N/A | N/A | N/A | Intervention: 30 (55)  Control: 26 (48) | N/A | N/A | Total: 322 (72.0) | N/A |
| **Metabolic syndrome** | N/A | Total: 52.0 (59.5) | N/A | N/A | N/A | N/A | N/A | N/A | N/A | N/A | N/A |
| **Dyslipidemia** | N/A | N/A | N/A | N/A | N/A | MeDi+EVOO: 72 (79.1)  MeDi+Nuts: 67 (76.1)  Control: 59 (66.3) | N/A | N/A | N/A | N/A | N/A |
| **Cardiovascular**  **disease** | N/A | N/A | Total: 611 (8.8) | N/A | N/A | N/A | N/A | N/A | N/A | N/A | N/A |
| **Depression** | N/A | N/A | N/A | 298 (11.4) | N/A | N/A | N/A | N/A | N/A | N/A | N/A |

Abbreviations: MeDi: Mediterranean diet; EVOO: extra virgin olive oil; N/A: not available.

**Table S5.** Risk of bias assessment of the included cohort studies.

| **Study ID** | **1. Representativeness of the exposed cohort** | **2. Selection of the non-exposed cohort** | **3. Ascertainment of exposure** | **4. Demonstration that outcome of interest was not present at start of study** | **5. Comparability of cohorts on the basis of the design or analysis** | **6. Assessment of outcome** | **7. Was follow-up long enough for outcomes to occur** | **8. Adequacy of follow up of cohorts** | **Overall score** |
| --- | --- | --- | --- | --- | --- | --- | --- | --- | --- |
| Berr et al. 2009 (3) | A | A | C | A | A,B | A | B | B | 7 |
| Fischer et al. 2018 (4) | A | A | C | A | A,B | A | A | B | 8 |
| Galbete et al. 2015 (5) | C | A | C | A | A,B | A | B | A | 6 |
| Psaltopoulou et al. 2008 (8) | A | A | C | A | A,B | A | A | B | 8 |

**Selection**

1) Representativeness of the exposed cohort

a) truly representative of the average _______________ (describe) in the community *

b) somewhat representative of the average ______________ in the community *

c) selected group of users e.g. nurses, volunteers

d) no description of the derivation of the cohort

2) Selection of the non-exposed cohort

a) drawn from the same community as the exposed cohort *

b) drawn from a different source

c) no description of the derivation of the non-exposed cohort

3) Ascertainment of exposure

a) secure record (e.g. surgical records) *

b) structured interview *

c) written self-report

d) no description

4) Demonstration that outcome of interest was not present at start of study

a) yes *

b) no

**Comparability**

1) Comparability of cohorts on the basis of the design or analysis

a) study controls for _____________ (select the most important factor) *

b) study controls for any additional factor * (This criteria could be modified to indicate specific control for a second important factor.)

**Outcome**

1) Assessment of outcome

a) independent blind assessment *

b) record linkage *

c) self-report

d) no description

2) Was follow-up long enough for outcomes to occur

a) yes (select an adequate follow up period for outcome of interest) *

b) no

3) Adequacy of follow up of cohorts

a) complete follow up - all subjects accounted for *

b) subjects lost to follow up unlikely to introduce bias - small number lost - > ____ % (select an adequate %) follow up, or description provided of those lost) *

c) follow up rate < ____% (select an adequate %) and no description of those lost

d) no statement

**Table S6.** Risk of bias assessment of the included cross-sectional studies.

| **Study ID** | **1. Representativeness of the sample** | **2. Sample size** | **3. Non-respondents** | **4. Ascertainment of the exposure (risk factor)** | **5. The subjects in different outcome groups are comparable, based on the study design or analysis. Confounding factors are controlled.** | **6. Assessment of the outcome** | **7. Statistical test** | **8. Overall score** |
| --- | --- | --- | --- | --- | --- | --- | --- | --- |
| Anastasiou et al. 2017 (1) | A | B | C | A | A,B | A | A | 8 |
| Bajerska et al. 2017 (2) | C | B | C | A | A,B | A | A | 7 |
| Valls-Pedret et al. 2012 (10) | A | B | C | A | A,B | A | A | 8 |
| Talhaoui et al. 2023 (11) | C | B | C | A | A,B | A | A | 7 |

**Selection**

1) Representativeness of the sample:

a) Truly representative of the average in the target population* (all subjects or random sampling)

b) Somewhat representative of the average in the target population* (non-random sampling)

c) Selected group of users

d) No description of the sampling strategy

2) Sample size:

a) Justified and satisfactory*

b) Not justified

3) Non-respondents:

a) Comparability between respondents and non-respondents characteristics is established, and the response rate is satisfactory*

b) The response rate is unsatisfactory, or the comparability between respondents and non-respondents is unsatisfactory

c) No description of the response rate or the characteristics of the responders and the non-responders

4) Ascertainment of the exposure (risk factor):

a) Validated measurement tool**

b) Non-validated measurement tool, but the tool is available or described*

c) No description of the measurement tool

**Comparability (maximum two stars)**

1) The subjects in different outcome groups are comparable, based on the study design or analysis. Confounding factors are controlled.

a) The study controls for the most important factor (select one). *

b) The study control for any additional factor. *

**Outcome (maximum three stars)**

1) Assessment of the outcome:

a) Independent blind assessment**

b) Record linkage**

c) Self report*

d) No description

2) Statistical test:

a) The statistical test used to analyze the data is clearly described and appropriate, and the measurement of the association is presented, including confidence intervals and the probability level (p value)*

b) The statistical test is not appropriate, not described or incomplete

**Table S7.** Risk of bias assessment of included randomized clinical trials.

| Study ID | D1. Bias arising  from the  randomization  process | D2. Bias due to  deviations from  intended  interventions | D3. Bias due to  missing  outcome data | D4. Bias in  measurement  of the outcome | D5. Bias in  selection of the  reported result | Overall bias |
| --- | --- | --- | --- | --- | --- | --- |
| Martínez-Lapiscina et al. 2013 (6) | Low Risk | Low Risk | Low Risk | Low Risk | Low Risk | Low Risk |
| Mazza et al. 2018 (7) | Some Concerns | High Risk | High Risk | Low Risk | Low Risk | High Risk |
| Tsolaki et al. 2020 (9) | Low Risk | Low Risk | Low Risk | Low Risk | Low Risk | Low Risk |

**RoB2 overall risk of bias judgment:**

Low risk of bias = The study is judged to be at low risk of bias for all domains for this result.

Some concerns = The study is judged to raise some concerns in at least one domain for this result, but not to be at high risk of bias for any domain.

High risk of bias = The study is judged to be at high risk of bias in at least one domain, or to have some concerns for multiple domains in a way that substantially lowers confidence in the result.

**References**

1. Anastasiou CA, Yannakoulia M, Kosmidis MH, Dardiotis E, Hadjigeorgiou GM, Sakka P, et al. Mediterranean diet and cognitive health: Initial results from the Hellenic Longitudinal Investigation of Ageing and Diet. PLoS One. 2017;12(8):e0182048.

2. Bajerska J, Woźniewicz M, Suwalska A, Jeszka J. Eating patterns are associated with cognitive function in the elderly at risk of metabolic syndrome from rural areas. European Review for Medical and Pharmacological Sciences. 2014;18(21):3234-45.

3. Berr C, Portet F, Carriere I, Akbaraly TN, Feart C, Gourlet V, et al. Olive oil and cognition: results from the three-city study. Dement Geriatr Cogn Disord. 2009;28(4):357-64.

4. Fischer K, Melo van Lent D, Wolfsgruber S, Weinhold L, Kleineidam L, Bickel H, et al. Prospective Associations between Single Foods, Alzheimer's Dementia and Memory Decline in the Elderly. Nutrients. 2018;10(7).

5. Galbete C, Toledo E, Toledo JB, Bes-Rastrollo M, Buil-Cosiales P, Marti A, et al. Mediterranean diet and cognitive function: The sun project. Journal of Nutrition, Health and Aging. 2015;19(3):305-12.

6. Martínez-Lapiscina EH, Clavero P, Toledo E, San Julián B, Sanchez-Tainta A, Corella D, et al. Virgin olive oil supplementation and long-term cognition: the PREDIMED-NAVARRA randomized, trial. J Nutr Health Aging. 2013;17(6):544-52.

7. Mazza E, Fava A, Ferro Y, Rotundo S, Romeo S, Bosco D, et al. Effect of the replacement of dietary vegetable oils with a low dose of extravirgin olive oil in the Mediterranean Diet on cognitive functions in the elderly. J Transl Med. 2018;16(1):10.

8. Psaltopoulou T, Kyrozis A, Stathopoulos P, Trichopoulos D, Vassilopoulos D, Trichopoulou A. Diet, physical activity and cognitive impairment among elders: the EPIC-Greece cohort (European Prospective Investigation in Cancer and Nutrition). PUBLIC HEALTH NUTRITION. 2008;11(10):1054-62.

9. Tsolaki M, Lazarou E, Kozori M, Petridou N, Tabakis I, Lazarou I, et al. A Randomized Clinical Trial of Greek High Phenolic Early Harvest Extra Virgin Olive Oil in Mild Cognitive Impairment: The MICOIL Pilot Study. J Alzheimers Dis. 2020;78(2):801-17.

10. Valls-Pedret C, Lamuela-Raventós RM, Medina-Remón A, Quintana M, Corella D, Pintó X, et al. Polyphenol-rich foods in the Mediterranean diet are associated with better cognitive function in elderly subjects at high cardiovascular risk. J Alzheimers Dis. 2012;29(4):773-82.

11. Talhaoui A, Aboussaleh Y, Bikri S, Rouim FZ, Ahami A. THE RELATIONSHIP BETWEEN ADHERENCE TO A MEDITERRANEAN DIET AND COGNITIVE IMPAIRMENT AMONG THE ELDERLY IN MOROCCO. Acta Neuropsychologica. 2023;21(2):125-38.
